# High Resolution T1ρ Mapping of *In Vivo* Human Knee Cartilage at 7T

**DOI:** 10.1371/journal.pone.0097486

**Published:** 2014-05-15

**Authors:** Anup Singh, Mohammad Haris, Kejia Cai, Feliks Kogan, Hari Hariharan, Ravinder Reddy

**Affiliations:** 1 CMROI, Department of Radiology, University of Pennsylvania, Philadelphia, Pennsylvania, United States of America; 2 Center for Biomedical Engineering, Indian Institute of Technology Delhi, Delhi, India; 3 Research Branch, Sidra Medical and Research Center, Doha, Qatar; 4 Radiology, University of Illinois at Chicago, Chicago, Illinois, United States of America; Delft University of Technology (TUDelft), Netherlands

## Abstract

**Purpose:**

Spin lattice relaxation time in rotating frame (T1ρ) mapping of human knee cartilage has shown promise in detecting biochemical changes during osteoarthritis. Due to higher field strength, MRI at 7T has advantages in term of SNR compared to clinical MR scanners and this can be used to increase in image resolution. Objective of current study was to evaluate the feasibility of high resolution T1ρ mapping of *in vivo* human knee cartilage at 7T MR scanner.

**Materials and Methods:**

In this study we have used a T1ρ prepared GRE pulse sequence for obtaining high resolution (in plan resolution  = 0.2 mm^2^) T1ρ MRI of human knee cartilage at 7T. The effect of a global and localized reference frequency and reference voltage setting on B_0_, B_1_ and T1ρ maps in cartilage was evaluated. Test-retest reliability results of T1ρ values from asymptomatic subjects as well as T1ρ maps from abnormal cartilage of two human subjects are presented. These results are compared with T1ρ MRI data obtained from 3T.

**Results:**

Our approach enabled acquisition of 3D-T1ρ data within allowed SAR limits at 7T. SNR of cartilage on T1ρ weighted images was greater than 90. Off-resonance effects present in the cartilage B_0_, B_1_ and T1ρ maps obtained using global shim and reference frequency and voltage setting, were reduced by the proposed localized reference frequency and voltage setting. T1ρ values of cartilage obtained with the localized approach were reproducible. Abnormal knee cartilage showed elevated T1ρ values in affected regions. T1ρ values at 7T were significantly lower (p<0.05) compared to those obtained at 3T.

**Conclusion:**

In summary, by using proposed localized frequency and voltage setting approach, high-resolution 3D-T1ρ maps of *in vivo* human knee cartilage can be obtained in clinically acceptable scan times (<30 min) and SAR constraints, which provides the ability to characterize cartilage molecular integrity.

## Introduction

Cartilage is a thin tissue with a thickness varying between 1 and 6 mm [1] and consists of multiple zones, particularly superficial, transitional or middle and deep zones. The superficial zone is the thinnest with a relative thickness of ∼10-20% and the deep zone is thickest with a thickness of ∼50–60% of the total cartilage thickness. It has been reported that Osteoarthritis (OA) starts in the superficial zone with loss of proteoglycans [2]–[4]. High resolution MRI is always desirable for better characterization of focal aberrations in cartilage molecular integrity. Recent anatomical imaging studies on musculoskeletal system at 7T whole body MRI scanner have already shown around two fold expected SNR advantage compared to 3T [5]–[7].

Spin lattice relaxation time in rotating frame (T1ρ) MRI [8] measurements have been used to explore incipient molecular changes associated with OA. Several T1ρ mapping studies of very high resolution in *ex vivo* cartilage tissue have shown exquisite cartilage classification [9]–[11]. T1ρ mapping has been used to characterize *in vivo* human articular cartilage at clinical MR field strengths (1.5 T and 3T) [9], [12]–[22]. At clinical field strengths (1.5 T and 3T), planar resolution of *in vivo* 3D-T1ρ maps has been limited due to a combination of issues related to adequate SNR, appropriate RF coils and scanning time constraints.

T1ρ MRI of knee cartilage at 7T is also expected to have same SNR advantage compared to 3T and to provide better classification of cartilage regional integrity during OA. The SNR gain can be exploited for obtaining higher resolution T1ρ mapping at 7T. However, T_1ρ_ MRI at 7T is challenging due to increase in specific absorption ratio (SAR) and B_0_ and B_1_ field inhomogeneity effects.

In this study, for the first time, we have obtained high resolution 3D-T1ρ weighted data and maps from human knee cartilage at 7T. A localized frequency and reference voltage setting approach for reducing off resonance effects is presented. The B_0_ and B_1_ field inhomogeneity maps of knee cartilage are obtained and SNR of human knee cartilage is computed from articular cartilage of all the subjects. Reproducibility of T_1ρ_ values in cartilage of asymptomatic subjects is tested. Finally, T_1ρ_ data from abnormal knee cartilage of two human subjects is presented. T_1ρ_ values of articular cartilage from healthy human subjects obtained from 7T are compared with those obtained at a 3T clinical scanner. Advantages and challenges of implementing T_1ρ_ mapping at 7T are outlined.

## Methods and Materials

### Ethics Statement

All Studies were conducted under an approved Institutional Review Board protocol of the University of Pennsylvania. Written informed consent from each volunteer was obtained after explaining the study protocol.

### Subjects, MRI Scanner and Coil Information

Eight healthy volunteers (20–35 Y), one volunteer (44 Y) with a previously diagnosed with meniscal tear and cartilage pathology and one volunteer with knee pain (62 Y) underwent T1ρ MRI of knee at a whole body 7T scanner (Magnetom 7 Tesla, Siemens-Healthcare, Erlangen, Germany) using a CP Transmit/28 channel receive array knee coil (inner diameter  = 15.4 to 18 cm, Quality Electrodynamics, Mayfield Village, OH). Subjects were scanned for either one or both knees. Some of the healthy subjects were scanned 3 to 4 times for optimization of protocol and evaluating field inhomogeneity effects.

### T1ρ Pulse Sequence

**Figure 1:**
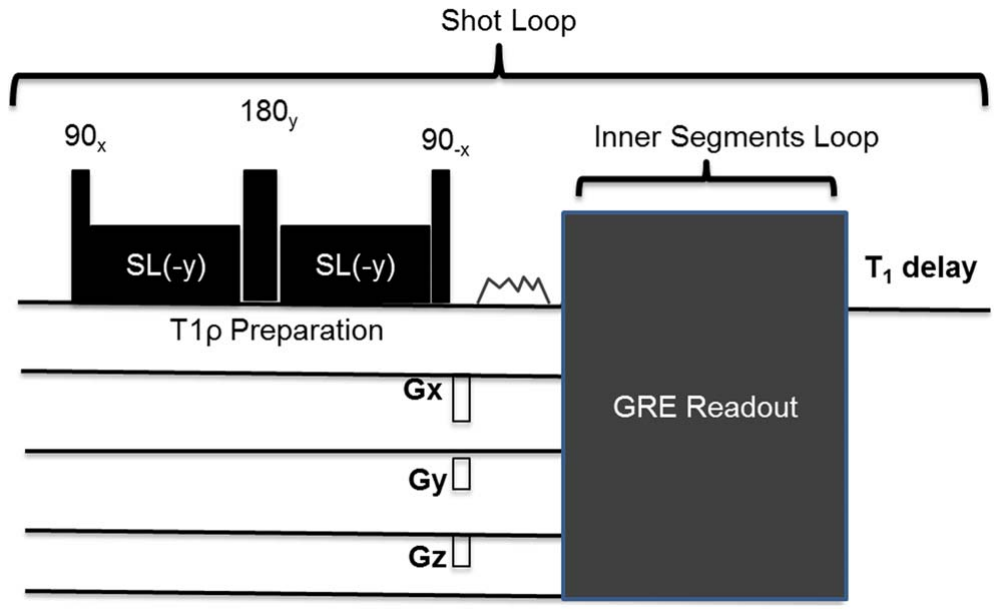
Single shot pulse sequence diagram of T1ρ MRI. T1ρ pulse sequence consist of a B_1_ and B_0_ compensated T1ρ preparation pulse cluster, crusher gradients, chemical shift selective fat saturation pulse, a gradient echo readout acquisition with centric phase encoding order and T1 recovery delay.

A modified T1ρ pulse sequence based on previously reported sequence [17], [23], consisting of a B_1_ and B_0_ compensated T1ρ preparation pulse cluster [17] followed by a chemical shift selective fat saturation pulse and a segmented radiofrequency spoiled gradient echo with multiple shots readout acquisition with centric phase encoding order was used (Figure 1).

### MRI Data Acquisition Procedure

T1ρ imaging protocol for knee cartilage consists of following steps. A tri-plane GRE localizer scans (∼0.5 min.); global shimming and center frequency and transmit voltage setting (∼1 min.); 3D structural image acquisition with isotropic voxel (0.6×0.6×0.6 mm^3^) used for guiding selection of T1ρ imaging slices (∼4 min); reference frequency and voltage were set corresponding to a small volume covering patellar cartilage using localized stimulated echo acquisition mode (STEAM) single voxel spectra (SVS) (∼2 min); 3D-T1ρ data in axial orientation for patellar cartilage (∼8 min); reference frequency and voltage were set corresponding to a small volume covering femoral and tibial cartilages using SVS (∼2 min); 3D-T1ρ data in coronal orientation for femoral and tibial cartilages (∼8 min).

### T1ρ MRI Data Protocol

3D T1ρ imaging was performed with spin lock pulse amplitude B_1sl_ = 500 Hz, spin lock times (TSL) = 0, 10, 20, 30, 40 ms and with imaging parameters: TR/TE = 9.7/4.9 ms, flip angle = 10^o^, FOV = 140×140×30 mm^3^, matrix size  = 448×224×10, number of averages  = 1, number of shots per slice encode  = 2 and a shot TR of 5 seconds. Scan time for one set of 3D-T1ρ data (slices  = 10 and TSLs  = 5) was 8.3 min. Two sets of T1ρ data were acquired, one in axial orientation (for patellar cartilage) and another in the coronal orientation (for weight-bearing femoral and tibial cartilage). Since higher resolution were required mainly along cartilage thickness, data were acquired with 50% phase encode resolution and the phase encoding directions were set as ‘right to left’ for both scans.

### B_0_ and B_1_ Data Protocol

For obtaining field inhomogeneity information on knee cartilage, B_0_ and B_1_ field maps were obtained for five healthy volunteers. For B_0_ map, we acquired WASSR data [24], [25] with following parameters: saturation B_1_ = 20 Hz and saturation duration  = 200 ms, saturation frequency offset range  = −0.8 to 0.8 ppm with step size of 0.1 ppm, TR/TE  = 7.8/3.9 ms, flip angle  = 10^o^, FOV = 140×140×30 mm^3^, matrix size  = 256×128×10, number of averages  = 1, number of shots per slice encode  = 1 and a shot TR of 5 seconds. For B_1_ field map, flip crush sequence with two flip angles 30 and 60 degree was used.

### Reproducibility Study Procedure

For reproducibility studies, knee was positioned in the approximately same place and orientation inside the coil by using foam pads. In addition, we used “ImScribe” software tool (written in Matlab) that allows reproducible selection of the same anatomical FOV in Siemens MRI. The program is written in Matlab and requires the installation of the SPM software toolbox. In this study, we have used affine transformation based registration in ImScribe tool. 3D structural imaging data from two scans and a localizer slice from first scan were used as an input in Imscribe software for obtaining same location during 2^nd^ scan. T1ρ data from the healthy subjects (n = 8) were obtained at two time points (different days, within 1 month period).

### T1ρ MRI Data at 3T

For comparison of T1ρ values, we also acquired T1ρ MRI data from six of the healthy volunteers with the same imaging sequence parameters in a 3T clinical scanner (Tim-Trio, Siemens-Healthcare, Erlangen, Germany) using a quadrature-spiral-birdcage transmit/8 channel receive Knee Coil (inner diameter  = 15.4 to 18 cm, In-Vivo, Gainesville, FL).

### Reference Voltage Calculation Using STEAM

The signal from SVS STEAM sequence with identical flip angles (α) for three pulses is proportional to 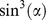. Flip angle *α* is directly proportional to reference voltage setting in the Siemens scanner, which corresponds to a peak B_1_ of 11.77 µT. We calculated the flip angle corresponding to a preset reference voltage (x) from maximum of water signals obtained with two spectra acquired with a reference voltage of x (S_1_) and 2x (S_2_), using the formula as: 
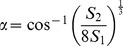
. Final reference voltage (V) is calculated using expression: 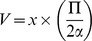.

### Image Processing

MRI data were processed using Image-J software [26] and in-house written programs in MATLAB. Data were pre-processed for motion correction using rigid body registration followed by segmentation of cartilage sections. 3D T1ρ data sets corresponding to each TSL (>0) were registered automatically with respect to TSL = 0 ms data. T1ρ-weighted (T1ρ-W) images corresponding to a TSL = 20 ms were used for generating 3D cartilage mask using manual segmentation of cartilage. Cartilage sections in 3D cartilage mask were further subdivided into medial and lateral sides and into three zones, deep, middle and superficial, by a semi-automatic segmentation program. This program requires the user input for medial and lateral side selection and it divides a 3D mask of cartilage into three regions (zones) using morphological operations. This division is just an approximation to expected cartilage division mentioned in the ‘Introduction’.

### SNR Calculations

The SNR of T1ρ-W images on cartilage was obtained, as a ratio of average value of a region of interest (ROI) on cartilage and standard deviation (s.d.) of an ROI in background noise[27], from T1ρ-W images. A factor of 0.655 was also multiplied to SNR for accounting Rician noise distribution in MRI magnitude images [27]. An ROI on the cartilage of central slice of T1ρ –W data and four ROIs in background noise of same slice were drawn and SNR of cartilage was computed with respect to each of these background noise ROIs. Final SNR was computed as an average of these four SNR. Average values and standard deviations were computed for multiple segmented sections. B_0_ and B_1_ (B_1rel_) maps were generated using previously described procedure in [24], [25] and [25]respectively.

### T1ρ Mapping

The T1ρ-W data corresponding to different TSLs were fitted voxel-wise to a mono-exponential decay expression, 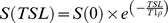, for computing T1ρ values. Goodness of fit parameter (R^2^) was also computed. In the current study, the T1ρ-W image corresponding to a TSL = 0 ms was used as a base or anatomical image. T1ρ maps were color overlaid on the base image.

### Statistical Analysis

Mean and s.d of T1ρ values in different cartilage facets were computed. For test-retest reliability experiment, Intraclass correlation (ICC) coefficient along with 95% confident interval was computed using SPSS (Version 20). ICC with p value less that 0.05 was considered as statistically significant. In addition, Pearson correlation coefficient and coefficient of variations were also computed. Student's t-test was used to evaluate significance of difference of T1ρ values in medial vs lateral side of cartilage at 7T. T1ρ values at 3T and 7T were also compared using t-test.

## Results

### SAR during T1ρ MRI

In this study, SAR was well within the scanner set limits in all the experiments. Moreover, none of the volunteers reported any heating discomfort during study.

### Reference Frequency Offset

In the current study, reference frequency obtained using a SVS covering patellar cartilage region at 7T was typically offset from the reference frequency obtained from scanner prescan by around 139±26 Hz (mean ± s.d.) in different experiments. For femoral and tibial cartilage this offset in frequency was 7±31 Hz (mean ± s.d.). After localized reference frequency setting, the offset variations obtained from B_0_ field maps across cartilage were around 0±30 Hz (mean ± s.d.).

### Reference Voltage Offset

Reference voltages obtained using SVS on different facets of articular cartilage and those obtained using scanner prescan were similar (within 6% difference). B_1rel_ map obtained after localized voltage setting showed a value of 1.0±0.1 (mean ± s.d.) on cartilage.

### SNR of T1ρ-W Images

The SNR of base images (In plane resolution = 0.2 mm^2^) on the cartilage averaged over all the subjects were ∼240, while the lowest SNR of T1ρ-weighted images (TSL = 40 ms) on the cartilage were ∼90 at 7T.

### Effect of SVS vs Scanner Prescan Based Reference Frequency and Voltage on T1ρ Mapping

**Figure 2:**
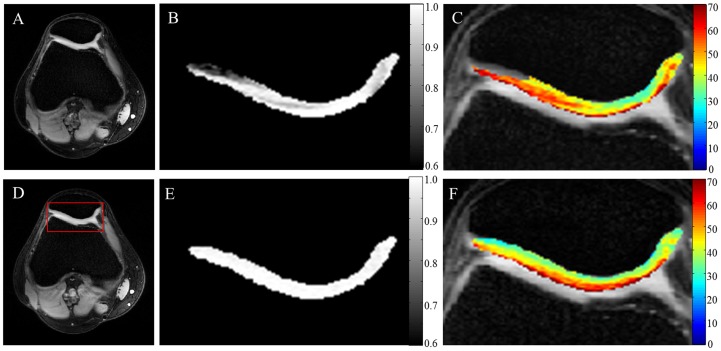
Off-resonance effect on T1ρ mapping. Top row contains anatomical image (A), goodness of fit (R^2^) map (B) and T1ρ map (C) corresponding to global volume reference frequency and voltage. Bottom row contains anatomical image (D), R^2^ map (E) and T1ρ map (F) corresponding to local volume (rectangular box on anatomical image) around patellar cartilage based reference frequency and voltage. Note that pixels on cartilage with R^2^<0.8 are not displayed on final map.

Quality of exponential fitting for T1ρ maps at 7T obtained using SVS based reference frequency and voltage were better compared to those obtained using scanner prescan as can be seen from corresponding R^2^ maps (Fig. 2B and 2E). Actual T1ρ map obtained with scanner prescan set reference frequency seems to show elevated T1ρ values in middle and deep zone of healthy cartilage as pointed by arrow on Fig. 2C. In all the experiments, localized reference and voltage setting approach resulted in R^2^ value greater than 0.9 for all the voxels in the cartilage and hence improved the reliability of high resolution T1ρ mapping.

### T1ρ Data from Healthy Subject

**Figure 3:**
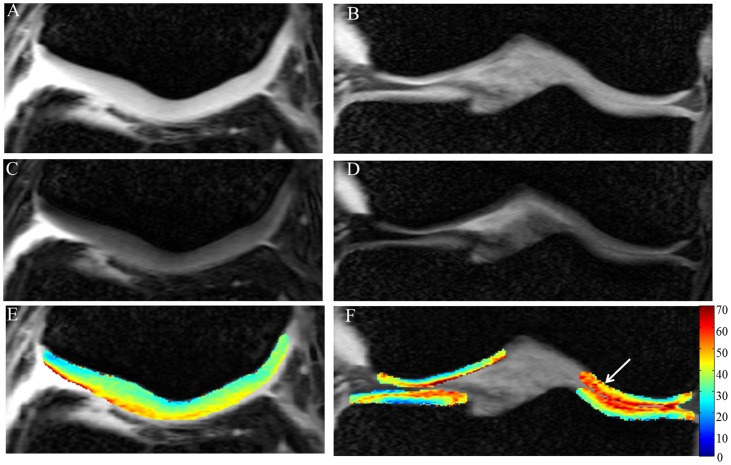
High resolution T1ρ maps of articular cartilage. First column images show patellar cartilage in axial orientation and 2^nd^ column images show femoral and tibial cartilage in coronal orientation of a healthy volunteer at 7T. First row (A&B) contain high resolution anatomical images (TSL = 0), 2^nd^ row (C&D) contain high resolution T1ρ-weighted images corresponding to TSL = 40 ms and 3^rd^ row contain high resolution T1ρ (ms) maps of cartilage overlaid on anatomical images. Arrow indicates the medial side femoral cartilage with reduced contrast among different layers due to magic angle effect. Note that these are cropped images.

T1ρ-W and T1ρ maps from articular cartilage of a healthy subject are shown in Fig.3. T1ρ values in most of the cartilage are higher in the superficial zone compared to the deep zone. This trend of T1ρ values from deep to superficial zone is maintained in most of the cartilage, except in a few regions where contrast among the layers disappears (as pointed out by arrow in Fig. 3E-F). This phenomenon has been characterized due to the magic angle effect, which decouples dipolar-dipolar interaction of collagen and eliminates contrast between layers [9].

### Reproducibility of T1ρ Mapping

**Figure 4:**
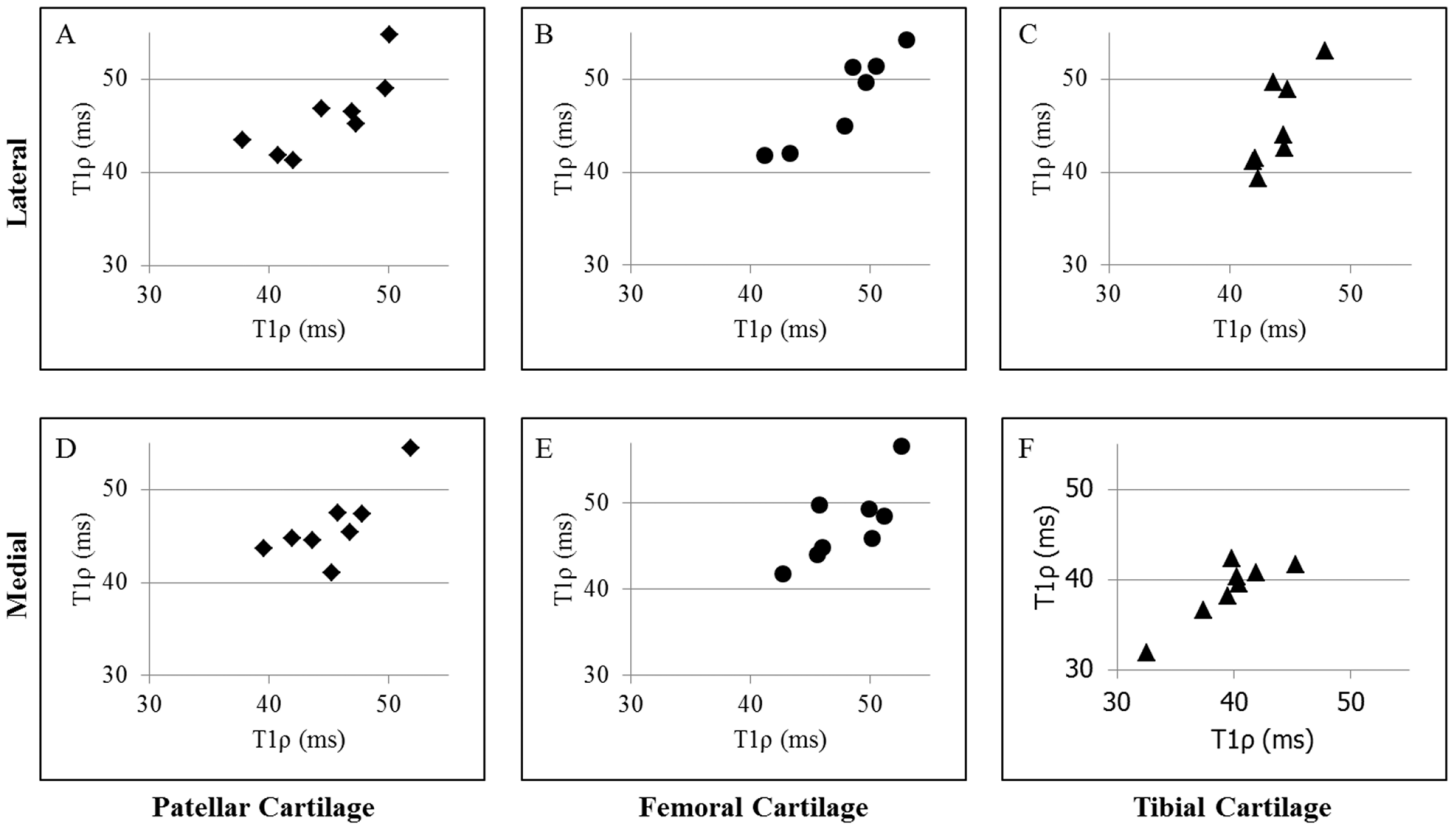
Scatter plots of test-retest data of T1ρ values in different cartilage facets for 8 subjects at 7T.

Scatter plot (Fig. 4) show T1ρ values in test-retest experiment. ICC coefficients, along with 95% CI, for test-retest reliability of T1ρ measurements in different facets of cartilage are reported in Table 1. ICC coefficients in all the facets of cartilage are statistically significant (p<0.05). Note that 95% CI in some facets are quite wide (Table 1). A high Pearson correlation coefficient was observed for test-retest reliability experiment (Table 2). Average coefficient of variation was under 5% in all the cartilage facets for test-retest experiment (Table 3).

**Table 1:** Intra class correlation (ICC) coefficient values for test-retest reliability experiment along with 95% CI in open brackets.

Cartilage	Patellar	Femoral	Tibial
Lateral	0.87(0.38, 0.97)	0.86(0.23, 0.97)	0.93(0.69, 0.99)
Medial	0.88(0.47, 0.98)	0.96(0.81, 0.99)	0.73(−0.3, 0.95)

ICC coefficients were statistically significant (p<0.05) for all the cartilage facets.

**Table 2:** Pearson correlation coefficient values for test-retest reliability experiment for medial and lateral sides of different cartilage facets.

Cartilage	Patellar	Femoral	Tibial
Lateral	0.87	0.86	0.93
Medial	0.88	0.96	0.73

Correlation coefficients were statistically significant (p<0.05) for all the cartilage facets.

**Table 3:** Coefficient variation (mean ± s.d.) values (%) for test-retest reliability experiment for medial and lateral sides of different cartilage facets.

Cartilage	Patellar	Femoral	Tibial
Lateral	3.6±2.4	3.5±2.2	2.5±1.9
Medial	3.5±3.2	2.3±1.6	4.3±3.2

### T1ρ Analysis

**Figure 5:**
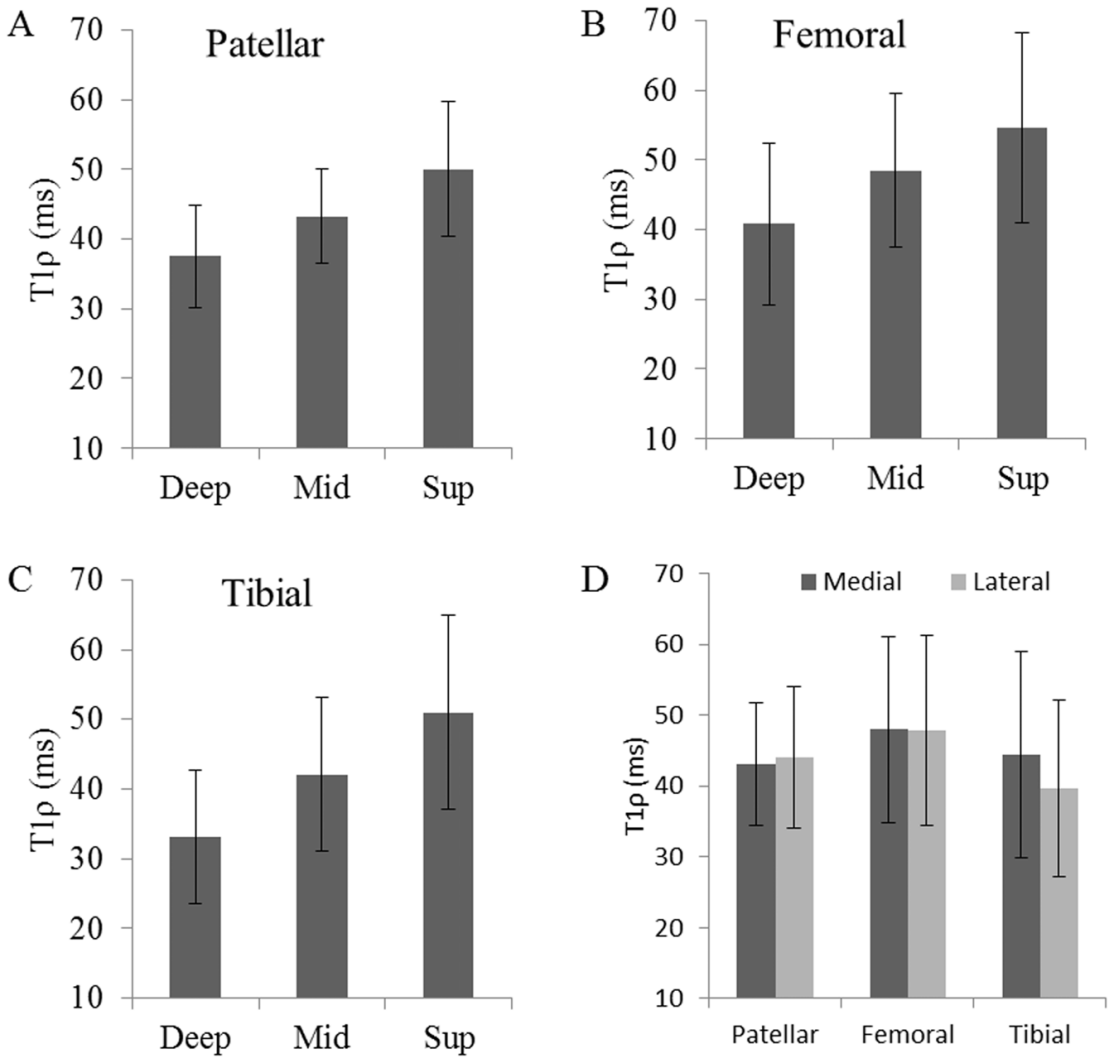
Bar plots represents T1ρ analysis of knee cartilage at 7T. T1ρ values from deep, middle and superficial zones of patellar, femoral and tibial cartilage are shown in Fig (A, B, C). T1ρ values from medial and lateral side of cartilage are shown in Fig. D.

T1ρ values in different facets of cartilage are shown in Fig. 5. T1ρ values were higher in superficial zones compared to middle and deep zones. For the data presented in current study, no significant difference was observed between medial and lateral sides of cartilage.

### T1ρ Analysis at 3T vs 7T

Average T1ρ values at 7T were lower (∼15%) compared to those obtained at 3T (Table 4). This difference was statistically significant (p<0.05) based upon t-test.

**Table 4:** Analysis of T1ρ values (ms) for human knee cartilage (n = 6) at 3T and 7T.

	Patellar	Femoral	Tibial
**3T**	50.5±2.4	49.3±3.4	47.2±2.1
**7T**	43.9±2.9	47.3±3.5	41.2±0.8

Values are reported as mean±s.d. calculated over all subjects data.

### T1ρ Data from Patients

**Figure 6:**
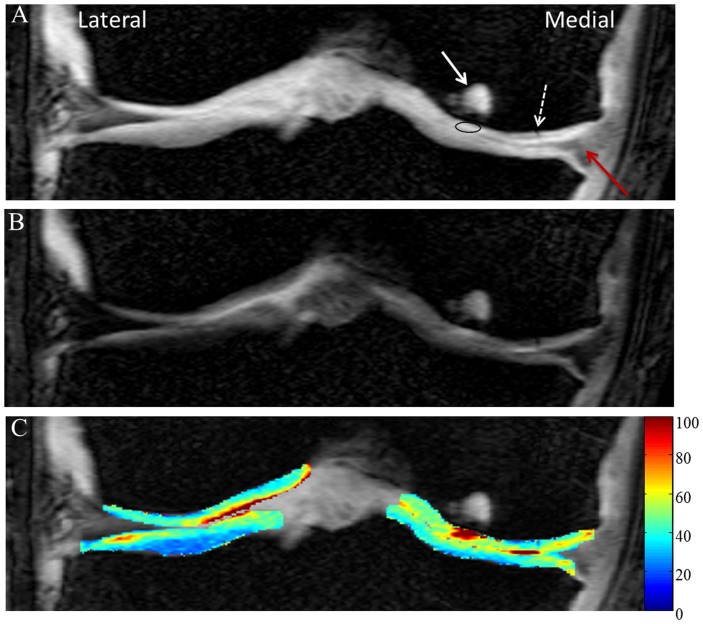
High resolution data from volunteer with meniscal tear and femoral cartilage pathology in medial side. Fig A represents anatomical image, Fig B represents T1ρ-weighted image corresponding to TSL = 40 ms and Fig C contain T1ρ (ms) map of femoral and tibial cartilages overlaid on anatomical image. The red arrow on anatomical image points to meniscal tear, the solid white arrow points to edema in femur and the dotted white arrow points to an unidentified pathology in cartilage. In the focal ROI (on anatomical image), mean T1ρ value is  = 113 ms. T1ρ values >300 ms are threshold to zero. Color scale was adjusted to highlight focal region on femoral cartilage with high T1ρ values. Note that only cropped images are shown.

**Figure 7:**
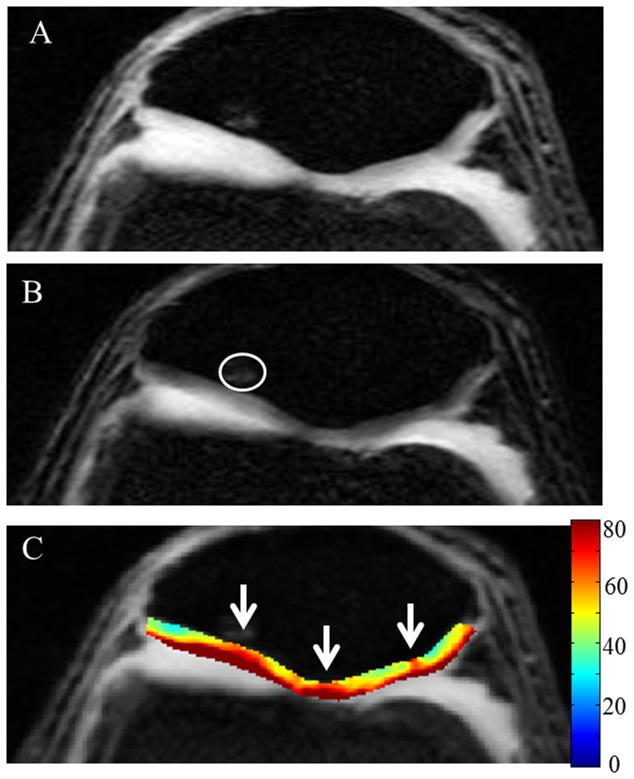
High resolution images from volunteer with abnormal patellar cartilage. Fig A represents anatomical image, Fig B represents T1ρ-weighted image corresponding to TSL  = 40 ms and Fig C contain T1ρ (ms) map of patellar cartilages overlaid on anatomical image. Circular ROI on Fig.B encircles edema in bone. White arrows on Fig.C point to abnormal cartilage regions. Color scale was adjusted to highlight abnormal region on cartilage with high T1ρ values. Note that only cropped images are shown.

Edema in femur bone and pathology in meniscus are clearly visible on high resolution anatomical image of a patient with knee injury (Fig. 6A). On the T1ρ-W image (Fig. 6B) signal intensity of femoral cartilage near femur bone edema is higher. In the medial femoral cartilage there is a clear elevation of T1ρ values in a focal region (Fig. 6C). Average T1ρ values in this focal ROI is 113 ms which is more than double compared to normal cartilage. Femoral cartilage thickness in this region is ∼1.5 mm. In addition, an unidentified pathology on femoral cartilage is clearly visible as a dark line on anatomical image (pointed by dotted white arrow on Fig. 6A). This pathology is observed on femoral cartilage of three consecutive slices. Results from another subject with abnormal patellar knee cartilage also showed elevated T1ρ values in the cartilage particularly in the three focal regions on patellar cartilage (Fig. 7).

## Discussion

In this study we have presented an approach for obtaining reliable high resolution 3D-T1ρ mapping of human knee cartilage at 7T MRI.

The effects of B_0_ and B_1_ inhomogeneities during spin-locking increased at 7T due to increase in field inhomogeneity. Offsets induced by B_0_ inhomogeneity results in off-resonance spin locking effects that produce inaccurate T1ρ maps. As such, a spin-echo based T1ρ pulse cluster is expected to mitigate B_0_-field inhomogeneity based artifacts, provided that the B_1_ field is homogenous. Because of B_1_ inhomogeneity in cartilage at 7T, spin echo based T1ρ pulse cluster may not be efficient in removing such artifacts. In this study, after global shimming, reference frequency and reference voltage from a local volume around cartilage was performed using localized SVS. With this approach, these field inhomogeneity artifacts were reduced significantly and reliable T1ρ maps of knee cartilage were obtained.

Standard Siemens scanner prescan uses a 10 mm axial slice at the isocenter of the magnet to set the reference frequency and voltage. In this study, femoral/tibial cartilage is located closer to isocenter than patellar cartilage. Hence the difference between SVS based reference frequency and scanner prescan based reference frequency was small, while in patellar cartilage this difference was higher. Scanner prescan based reference frequency setting leads to bigger off resonance effects in the T1ρ weighted images and results in erroneous T1ρ estimation.

Entire 3D-T1ρ imaging protocol as described in the method section can be run under 30 min with an in-plane resolution of 0.2 mm^2^. In this study we have presented T1ρ results using linear fitting approach only due to computing time efficiency. As such for some data sets we have compared T1ρ maps obtained using linear and non-linear fitting approaches. Due to high SNR of the T1ρ data in the current study, both approaches provided similar T1ρ values.

T1ρ values of femoral cartilage on the medial side of the patient with a meniscal tear are much higher compared to average T1ρ values from healthy cartilage. Since the femoral cartilage thickness in this region is ∼1.5 mm, higher resolution images and maps provided better classification of this focal region. Moreover, there is fluid in between the femoral and tibial cartilage in certain regions which have been segmented out. In the case of lower resolution, effects of partial voluming of cartilage with this free fluid would be increased and could confound interpretation of results. Although, free water suppression could reduce this problem to some extent it can also affect the cartilage signal differently in different zones. High resolution T1ρ mapping is essential in detecting changes in cartilage in such situations.

The main focus of our projects has been T1ρ imaging of patellar, femoral and tibial cartilages. In the current study, we have acquired separate data in axial and coronal orientations, as in our ongoing studies at lower field scanners. The main advantage of acquiring data in this mode is that we can reduce phase resolution without affecting the resolution along the thickness of the cartilage by setting the phase encoding direction as right-to-left. The overall scan time for sagittal orientation for covering patellar cartilage and weight-bearing femoral/tibial cartilage together, will require much longer scan times.

When using a GRE readout, T1 recovery process can mix with T1ρ preparation and can alter actual T1ρ values. For minimizing this effect we have used a centric encoding scheme and 2 shots. Inclusion of more shots may further reduce this effect but requires a longer scanning time.

Main source of contrast between cartilage layers is the dipolar-dipolar interaction of collagens. “Magic angle effects” can mitigate dipolar-dipolar interaction and hence reduce contrast between layers. This phenomenon was observed in some parts of cartilage where T1ρ contrast between deep and superficial zones was reduced substantially. Magic angle effects can interfere with interpretation of T1ρ values in cartilage. Alternatively, dipolar-dipolar interaction based effects can be mitigated using a high power spin lock (>1000 Hz); however, this is limited in human studies by SAR constraints.

Although in this study we demonstrated the T1ρ maps with 0.2 mm^2^ in plane resolution, it is possible to obtain even higher resolution images (<0.1 mm^2^), albeit with increased scan time that may be prohibitively too long to be useful for human studies. Therefore, the resolution of T1ρ maps in this study has to be traded for the scan-time savings.

In this study, we have used maximum TSL of 40 ms so that all the T1ρ experiments run under allowed systems SAR limits. Since T1ρ values in the healthy cartilage were below 60 ms, TSLs range used in the current study is sufficient to provide accurate estimation of T1ρ values. In case of increased T1ρ values as observed during cartilage pathology, estimation of T1ρ values may not be accurate due to limited TSLs range.

Reduction in T1ρ values at 7T compared to at 3T could be due to the increase in both chemical exchange based and dipolar-dipolar interaction based effects at 7T compared to 3T. By using higher spin lock power (>1000 Hz), these effects can be decoupled. However, spin lock power used in the current study is not sufficient to completely decouple these effects and this could have resulted in lower T1ρ values at 7T compared to 3T.

In this study, we have exploited the experimental high SNR ≥90 at 7T for obtaining high resolution 3D T1ρ maps of human knee cartilage. Due to around two fold increase in SNR from 3T to 7T, obtaining same resolution 3D T1ρ data with similar SNR at 3T, it would require around four times increase in scan time compared to 7T. Note that we have not compared the SNR at 7T with 3T due to the difference in the coils used in this study.

In conclusion, in the current study, we have demonstrated the feasibility of reliable T1ρ mapping and SNR advantage at 7T MRI was exploited to obtain high-resolution T1ρ maps of *in vivo* human knee cartilage in a clinically relevant scan times and SAR constraints. Feasibility of T1ρ MRI at 7T provides the ability to characterize spatial abnormalities in cartilage molecular integrity.
